# Evidence for increased thermogenesis in female C57BL/6J mice housed aboard the international space station

**DOI:** 10.1038/s41526-021-00150-y

**Published:** 2021-06-18

**Authors:** Carmen P. Wong, Urszula T. Iwaniec, Russell T. Turner

**Affiliations:** 1 Skeletal Biology Laboratory, School of Biological and Population Health Sciences, Oregon State University, Corvallis, OR USA; 2 Center for Healthy Aging Research, Oregon State University, Corvallis, OR USA

**Keywords:** Biochemistry, Molecular biology

## Abstract

Sixteen-week-old female C57BL/6J mice were sacrificed aboard the International Space Station after 37 days of flight (RR-1 mission) and frozen carcasses returned to Earth. RNA was isolated from interscapular brown adipose tissue (BAT) and gonadal white adipose tissue (WAT). Spaceflight resulted in differential expression of genes in BAT consistent with increased non-shivering thermogenesis and differential expression of genes in WAT consistent with increased glucose uptake and metabolism, adipogenesis, and β-oxidation.

Mice are facultative daily heterotherms and, in contrast to humans who maintain a near constant core body temperature over a wide range of environmental temperatures, mice can experience dramatic transient reductions in core temperature (torpor) when exposed to temperatures below thermoneutral^1,2^. Because of their small mass, mice are much more dependent on shivering and non-shivering thermogenesis for maintaining core body temperature than larger animals^3^.

The recommended temperature for housing mice in a laboratory setting (20–26 °C) is well below their thermoneutral zone, which depending upon strain, sex and age typically ranges from 29–31 °C^4^. Subthermoneutral housing induces adaptive responses in mice to increase generation of heat^5^. These adaptations include shivering thermogenesis, non-shivering thermogenesis, thermic effect of increased food consumption and physical activity. Sympathetic nervous system (SNS) neurotransmitters (norepinephrine and epinephrine) and the adipokine leptin are key factors involved in regulation of adaptive thermogenesis^6,7^. Subthermoneutral-housed mice use a variety of physiological and behavioral strategies to decrease their requirements for adaptive thermogenesis. These include entering torpor to lower core body temperature, and nest building, huddling and postural changes to minimize heat loss when exposed to a cool environment^8,9^.

The physiological demands required for successful adaptation to subthermoneutral housing by mice are considerable; for example, female mice housed at room temperature (22 °C) consumed 40% more food to achieve comparable weight gain and expressed 5-fold higher *Ucp1* gene expression in BAT (non-shivering thermogenesis) compared to thermoneutral-housed (32 °C) mice^10^. UCP-1 protein uncouples oxidative phosphorylation to produce heat instead of ATP. Extensive research supports the conclusion that by increasing UCP-1 protein levels, sympathetic signaling-driven non-shivering thermogenesis plays an important role in cold-induced thermoregulation in mice.

We hypothesize that spaceflight reduces the ability of mice to employ some of the strategies used to minimize adaptive thermogenesis, such as huddling and postural adjustments, resulting in an increased dependence on adaptive thermogenesis to maintain core body temperature. Group housing attenuates the increase in non-shivering thermogenesis in BAT in mice exposed to a cool environment^11^ but it is plausible that another aspect of the spaceflight environment increases sympathetic signaling, an important positive regulator of thermogenesis. Whatever the precise mechanism, increased thermogenesis is important because it influences multiple physiological processes^12–15^. Collateral changes associated with increased thermogenesis include cancellous bone loss, immune suppression, increases in glucocorticoid production, increases in blood pressure and heart rate, and altered tumor and tissue response to ionizing radiation^16–18^. Mechanistically, at least some of these responses are mediated by increased sympathetic outflow^14,19,20^.

Thus, activation of adaptive thermogenesis in mice housed in microgravity may introduce unrecognized and uncontrolled for confounding variables into spaceflight studies. We tested the hypothesis that nonshivering thermogenesis is increased in mice during spaceflight by measuring the effect of spaceflight on expression of genes related to energy metabolism in BAT and WAT in female C57BL/6J (B6) mice. In contrast to most prior spaceflight studies where flight animals were returned to Earth, the animals in this experiment (RR-1) were sacrificed aboard the International Space Station (ISS), avoiding the influence of restoration of normal gravitational loading.

Temperatures during the spaceflight mission ranged from a low of 21.3 °C to a high of 28.0 °C. The average housing temperatures within the Habitats enclosing the mice aboard ISS were 26.0 °C and 26.4 °C for flight and ground control animals, respectively. These housing temperatures, while above temperatures commonly used to house mice, are below thermoneutral for this species. Weight gain did not differ, but activity levels were higher in the flight animals, as was food and water depletion^21^, findings consistent with increased adaptive thermogenesis.

The effect of the spaceflight environment on differential expression of genes related to energy metabolism in BAT in flight animals is shown in Table 1. Transcript abundance of 13/84 genes were significantly altered in flight animals compared to ground controls. In particular, mice housed aboard ISS had 1.5x higher levels of *Ucp-1* in BAT, providing direct evidence for elevated non-shivering thermogenesis. Several genes associated with adipogenesis and/or thermogenesis, including *Adipoq*, *Ppargc1a, Cdkn1a*^22^, and *Cfd* were differentially expressed in BAT during spaceflight^23^. However, these changes may reflect adaptation to long duration spaceflight. Regardless of the mechanisms for initiation, increased *Ucp1*-mediated thermogenesis in BAT may have had a major impact on adipose tissue turnover in WAT (Table 2). Transcript abundance of 30/84 genes were significantly altered in flight animals compared to ground controls. Notable changes, including higher expression levels for (1) *Acacb* (Acetyl-CoA carboxylase), a key gene in regulation of fatty acid oxidation, (2) *Dio2* (Type II iodothyronine deiodinase), a key regulator of thyroid hormone action, (3) *Sic2a4* (Glut 4), an insulin-regulated glucose transporter, and (4) *Fasn* (fatty acid synthase) which catalyzes the synthesis of palmitate, were observed in WAT of flight animals. There was also evidence for induction of *Ucp-1*; expression level for this gene was very low (Ct > 30) in ground control mice and consistently detected in flight mice (Ct < 30), suggesting browning of WAT^24^. We conclude from these findings that, in spite of comparable housing temperatures, adaptive thermogenesis is increased in BAT of mice housed aboard the ISS compared to ground controls. This is important because increased thermogenesis may exaggerate (e.g., bone loss) or alter (e.g., response to ionizing radiation) physiological responses to spaceflight in mice. Because of species specific differences in thermoregulation, this could impact the translatability of the animal studies to astronauts.

**Table 1:** Gene array showing fold changes for differentially expressed genes in interscapular brown adipose tissue (BAT) in mice sacrificed aboard the International Space Station after 37 days of flight (RR-1 mission) compared to ground controls.

Space flight vs. ground control					
Symbol	Fold change	*P*<	Symbol	Fold change	*P*<
*Acacb*	−1.2	0.350	*Klf4*	−1.3	0.443
*Adig*	−1.1	0.599	*Lep*	−1.1	0.603
***Adipoq***	−**1.4**	**0.030**	*Lipe*	−1.1	0.347
*Adrb2*	−1.1	0.703	*Lmna*	−1	0.909
*Agt*	−1	0.919	*Lpl*	−1	0.988
*Angpt2*	−1.1	0.695	*Lrp5*	−1	0.715
*Axin1* ^***b***^	−1.2	0.101	*Mapk14*	−1	0.776
*Bmp2* ^***a***^	−1.2	0.160	*Ncoa2*	−1.1	0.182
*Bmp4* ^***b***^	1.2	0.296	*Ncor2*	1.2	0.195
*Bmp7*	1.1	0.412	*Nr0b2*	−1.3	0.229
*Ccnd1*	−1	0.983	*Nr1h3*	1.1	0.334
***Cdk4***	−**1.5**	**0.003**	***Nrf1***	−**1.4**	**0.029**
***Cdkn1a***	**2.4**	**0.002**	*Ppara*	−1.7	0.073
*Cdkn1b*	1.1	0.350	*Ppard*	−1.5	0.064
*Cebpa*	−1.1	0.261	*Pparg*	1	0.786
*Cebpb*	1	0.911	***Ppargc1a***	−**2**	**0.004**
*Cebpd*	1.2	0.368	*Ppargc1b*	1.2	0.194
***Cfd***	**2.2**	**0.007**	*Prdm16*	−1	0.773
*Creb1*	−1.1	0.168	*Rb1*	−1	0.863
*Ddit3*	1.2	0.223	*Retn*	−1.4	0.066
*Dio2* ^***b***^	−1.2	0.507	*Runx1t1*	−1.3	0.081
*Dkk1*	1.4	0.509	*Rxra* ^***a***^	−1	0.528
*Dlk1*	1.2	0.411	*Sfrp1*	1.3	0.156
*E2f1*	−1.5	0.108	*Sfrp5* ^***b***^	1.3	0.347
*Egr2*	−1.6	0.118	*Shh*	−1.4	0.186
*Fabp4*	−1	0.727	*Sirt1*	−1.1	0.441
*Fasn*	−1.1	0.715	*Sirt2*	−1	0.565
***Fgf1*** ^***b***^	**1.6**	**0.021**	*Sirt3*	−1.2	0.123
*Fgf10*	−1.3	0.191	*Slc2a4* ^***a***^	1.1	0.639
***Fgf2***	**1.5**	**0.050**	*Src*	1	0.665
*Foxc2*	−1.8	0.079	*Srebf1*	1.2	0.215
*Foxo1*	1	0.760	***Taz***	−**1.3**	**0.004**
*Gata2*	−1.2	0.139	***Tcf7l2***	**1.3**	**0.036**
*Gata3*	−1.3	0.609	***Tsc22d3***	**1.2**	**0.048**
*Hes1*	1.1	0.609	*Twist1*	−1.1	0.446
*Insr*	−1	0.524	***Ucp1***	**1.5**	**0.021**
*Irs1*	−1.5	0.051	*Vdr* ^***b***^	−1.2	0.098
***Irs2***	−**2**	**0.019**	*Wnt1* ^***b***^	−1.3	0.351
*Jun*	−1.5	0.114	*Wnt10b* ^***b***^	−1.3	0.147
*Klf15*	−1.1	0.598	*Wnt3a*	−1.5	0.107
*Klf2*	−1.2	0.159	*Wnt5a*	1	0.944
*Klf3*	−1.2	0.063	*Wnt5b*	1.3	0.290

^a^This gene’s average relative expression is low in control (Ct > 30) and reasonable in flight sample (Ct < 30).

^b^This gene’s relative expression level is low in both control and flight samples (Ct > 30).

**Table 2:** Gene array showing fold changes for differentially expressed genes in gonadal white adipose tissue (WAT) in mice sacrificed aboard the International Space Station after 37 days of flight (RR-1 mission) compared to ground controls.

Space flight *vs.* ground control					
Symbol	Fold change	*P*<	Symbol	Fold change	*P*<
***Acacb***	**4**	**0.001**	*Klf4*	1.1	0.291
***Adig***	**1.4**	**0.014**	*Lep*	−1.4	0.662
*Adipoq*	−1.1	0.414	*Lipe*	1	0.932
*Adrb2* ^*a*^	1.5	0.107	***Lmna***	**1.3**	**0.042**
*Agt*	1.1	0.635	*Lpl*	−1.2	0.145
*Angpt2*	−1.5	0.102	*Lrp5*	1.1	0.518
*Axin1*	1.5	0.117	***Mapk14***	**1.7**	**0.002**
***Bmp2***	**2.9**	**0.020**	*Ncoa2*	1.3	0.102
*Bmp4* ^*a*^	−1.2	0.945	*Ncor2*	1.3	0.480
***Bmp7***	**3.5**	**0.030**	***Nr0b2***	**2.4**	**0.048**
***Ccnd1***	**1.8**	**0.008**	***Nr1h3***	**1.5**	**0.001**
*Cdk4*	1.2	0.212	***Nrf1***	**2.1**	**0.046**
***Cdkn1a***	**3**	**0.001**	*Ppara* ^*a*^	1.7	0.121
*Cdkn1b*	1.3	0.104	***Ppard*** ^*a*^	**2**	**0.003**
*Cebpa*	1	0.708	***Pparg***	**1.3**	**0.031**
***Cebpb***	**2.2**	**0.003**	*Ppargc1a*	2.1	0.098
*Cebpd*	1.3	0.193	***Ppargc1b***	**2.4**	**0.003**
***Cfd***	**2.3**	**0.002**	*Prdm16*	2.2	0.063
***Creb1***	**1.8**	**0.003**	*Rb1*	1.1	0.095
*Ddit3*	1.3	0.102	***Retn***	**1.6**	**0.046**
***Dio2***	**5.5**	**0.042**	***Runx1t1*** ^*a*^	**3.2**	**0.009**
*Dkk1* ^*b*^	3.5	0.108	*Rxra*	1.6	0.067
*Dlk1* ^*b*^	2.8	0.077	*Sfrp1* ^*a*^	−1	0.957
*E2f1*	1.7	0.106	*Sfrp5* ^*a*^	1.3	0.709
*Egr2* ^*b*^	1.8	0.194	***Shh***	**5**	**0.014**
*Fabp4*	1.2	0.094	*Sirt1*	1.5	0.072
***Fasn***	**2.5**	**0.009**	***Sirt2***	**1.2**	**0.049**
*Fgf1*	1.3	0.840	***Sirt3***	**2**	**0.033**
*Fgf10*	1.1	0.516	***Slc2a4***	**2.2**	**0.002**
*Fgf2*	−1.1	0.872	***Src*** ^*a*^	**2.2**	**0.018**
*Foxc2* ^*b*^	2	0.102	***Srebf1***	**2.5**	**<0.001**
***Foxo1***	**1.3**	**0.040**	*Taz*	1.5	0.051
*Gata2*	2.4	0.064	***Tcf7l2***	**1.6**	**0.007**
*Gata3*	4.5	0.079	*Tsc22d3*	−1	0.726
*Hes1* ^*a*^	2.7	0.102	***Twist1***	**1.6**	**0.022**
*Insr*	1.3	0.089	*Ucp1* ^*a*^	29.3	0.122
*Irs1*	1	0.881	*Vdr* ^*b*^	3	0.145
*Irs2*	−1.3	0.400	*Wnt1* ^*b*^	2.9	0.080
*Jun*	−1.6	0.132	*Wnt10b* ^*b*^	4.1	0.210
*Klf15*	−1.2	0.329	*Wnt3a* ^*b*^	4.2	0.075
*Klf2*	−1.2	0.186	*Wnt5a*	1.9	0.052
*Klf3*	1.1	0.752	*Wnt5b* ^*b*^	2.2	0.112

^a^This gene’s average relative expression is low in control (Ct  <  30) and reasonable in flight sample (Ct < 30).

^b^This gene’s relative expression level is low in both control and flight samples (Ct > 30).

## Methods

### Spaceflight study

Details of the spaceflight mission are published^25^. Animal protocol was reviewed and approved by the NASA Institutional Animal Care and Use Committee prior to the conduct of experiments. In brief, 16-week-old female B6 mice were sacrificed aboard the ISS after 37 days of flight and frozen carcasses were returned to Earth for tissue preparation and method validation as described^21^. Sensors in the Habitats (flight and ground) monitored and relayed information including component temperature and humidity. The data were logged at sampling rate of 1 Hz. Ground control mice were sacrificed and processed using the same timelines and protocols as the flight animals.

### Gene expression study

RNA was isolated from individual BAT (*n* = 8/group) and WAT (*n* = 6/group) samples. RNA integrity number (RIN) was assessed using an Agilent Bioanalyzer (Santa Clara, CA, USA). A RIN value of 5 and above is required to ensure reliable quantification of gene expression by RT-qPCR^26,27^. The RIN numbers (mean ± SE, *n* = 6/group) for RNA isolated from BAT from flight and ground control animals were 6.55 ± 0.47, and 7.42 ± 0.46, respectively. Thus RNA from both groups of animals were of good quality. mRNA was reverse transcribed into cDNA using SuperScript III First-Strand Synthesis SuperMix for qRT-PCR (ThermoFisher Scientific). Expression levels for genes related to adipogenesis was determined for BAT and WAT using the Mouse Adipogenesis RT^2^ Profiler PCR Array (Qiagen). Gene expression was normalized using GusB and ActB housekeeping genes, and relative quantification (ΔΔ Ct method) was determined using RT^2^ Profiler PCR Array Data Analysis software (Qiagen).

### Reporting summary

Further information on research design is available in the Nature Research Reporting Summary linked to this article.

## Supplementary information

Supplementary Information

Reporting Summary

## Data Availability

Data are available in Supplemental Table 1 for BAT PCR array and Supplemental Table 2 for WAT PCR array.
