# Supplementary material for: Evidence for increased thermogenesis in female C57BL/6J mice housed aboard the international space station
Source: NPJ Microgravity. 2021 Jun 18;7:23. doi: 10.1038/s41526-021-00150-y (PMC8213760; doi:10.1038/s41526-021-00150-y)
Supplement: Supplementary file 1 — Supplementary Information [file 41526_2021_150_MOESM1_ESM.pdf]

Supplemental Table 1: BAT adipogenesis PCR array Ct values

Well	Symbol	Ground ctl #31	Ground ctl #32	Ground ctl #34	Ground ctl #35	Ground ctl #36	Ground ctl #37	Ground ctl #38	Ground ctl #39	Flight #21	Flight #22	Flight #23	Flight #25	Flight #27	Flight #28	Flight #29	Flight #30
A01	Acacb	22.209913	21.872292	22.13289	20.644073	21.581562	20.700611	21.409292	22.021988	23.689121	23.785892	22.37419	21.241447	21.054394	23.56562	22.047813	21.11307
A02	Adig	20.782305	20.559107	20.767958	19.749664	20.511457	19.687819	20.262594	20.522446	21.982733	21.741722	20.895395	20.062555	19.991991	22.34419	20.953098	20.245945
A03	Adipoq	21.057589	20.531223	20.79451	19.386633	20.289986	19.441517	19.982288	20.574413	22.772314	22.603144	20.62761	20.341152	20.00931	22.984196	20.996368	20.355923
A04	Adrb2	27.407274	27.155195	27.529417	26.97949	25.476152	26.783468	26.382847	26.33455	27.965284	28.317678	25.979382	26.894403	27.238148	28.481596	27.562943	26.953382
A05	Agt	29.252787	28.333569	28.445286	26.618855	28.730034	27.723194	28.170507	31.016344	29.69368	31.873653	27.025692	28.333994	28.189327	30.330297	28.707315	28.899708
A06	Angpt2	27.605677	27.704245	27.716066	27.302788	27.328384	27.179373	27.134058	27.503881	28.839012	29.065193	27.805626	28.405504	27.485243	28.127703	27.854866	27.805363
A07	Axin1	26.50087	26.17136	26.454142	25.382263	26.50485	25.597956	26.116798	26.49928	28.161112	28.711002	26.8746	26.169535	25.957626	27.844818	26.638838	25.795353
A08	Bmp2	31.139977	31.291712	30.960377	30.088373	31.64786	30.843122	30.748285	31.30347	32.795025	32.831562	31.527107	31.291151	31.090235	32.29715	31.777035	30.96084
A09	Bmp4	30.310894	29.68715	30.073069	29.429335	30.106424	29.270897	30.081831	29.397484	30.578342	31.375265	28.43953	29.672747	29.770485	31.857346	29.939636	29.558525
A10	Bmp7	30.569036	30.854998	30.956758	29.442316	30.609806	30.550365	30.588758	29.911516	31.519619	31.929829	29.99949	30.534668	30.522905	31.267622	30.754965	30.527266
A11	Ccnd1	25.490545	26.150568	26.461641	25.557995	25.83042	25.987335	26.307447	25.358627	26.636887	27.685621	25.584822	26.216469	25.8499	27.337013	26.324718	26.352633
A12	Cdk4	24.926027	24.496778	24.784124	23.455036	24.724436	23.789051	24.190126	24.879835	26.571413	26.833025	25.218243	24.584383	24.600254	26.746058	25.199066	24.660566
B01	Cdkn1a	23.61973	22.992208	22.74037	22.521673	23.47508	23.073904	23.627295	23.960955	24.193996	24.46457	22.785698	21.233202	20.896847	22.903122	21.541042	22.334171
B02	Cdkn1b	26.41056	26.01307	26.4518	25.812344	26.380825	26.515001	26.269222	26.889383	27.582521	28.002594	26.20592	26.573618	26.095646	26.699472	26.335861	26.404852
B03	Cebpa	24.408804	24.232416	24.458487	23.448734	24.471073	24.16028	24.226154	26.471098	25.699701	26.16399	24.857193	24.58224	24.12287	26.60468	24.74673	24.636826
B04	Cebpb	24.822916	24.985895	24.750628	24.349733	25.61936	25.076664	24.948515	26.575014	26.62903	26.342182	25.396517	24.527445	24.917582	26.677973	25.68828	25.093157
B05	Cebpd	30.18498	29.59572	29.297617	28.35174	29.8184	29.066282	29.199501	30.38851	31.189383	31.434046	27.37366	28.996977	29.495388	30.397497	29.474012	29.73278
B06	Cfd	18.062191	18.754765	18.717949	17.98731	18.179274	18.384089	18.041172	17.812376	17.62374	18.036798	16.627583	17.62565	16.933504	18.892374	17.724394	18.128532
B07	Creb1	26.444283	26.289375	26.488827	25.477238	26.701506	26.007383	26.374	26.313953	27.741886	28.419353	26.431341	26.301706	26.082151	27.506468	26.96285	26.476147
B08	Ddit3	24.301043	23.901356	24.192808	23.180885	23.714165	23.104435	23.843275	24.08851	24.835377	25.140537	24.654331	23.77528	23.195938	24.14736	23.668118	23.748777
B09	Dio2	23.115538	23.92497	24.36806	22.83242	23.506031	22.459885	23.665821	24.233454	25.351107	25.305159	25.8634	22.764812	22.718285	25.06033	24.820196	23.312326
B10	Dkk1	31.877668	34.70372	36.88884	35.719624	37.54668	34.137417	32.56487	32.37537	35.37446	39.189793	33.816235	33.827183	34.137917	31.75016	32.88932	Undetermined
B11	Dlk1	27.802011	29.232004	27.344606	26.74101	27.837214	27.483438	27.805971	28.76586	27.819633	29.332325	28.231022	28.247658	27.622154	28.464417	27.37711	28.247307
B12	E2f1	26.035778	25.796375	25.78216	23.683176	25.248035	24.330507	24.771244	27.279493	28.438084	28.467081	26.489761	25.160217	25.158552	27.276644	25.312557	25.781042
C01	Egr2	27.918402	26.651169	26.777273	24.73903	26.331137	25.763247	25.825182	29.168734	30.84374	29.944065	27.615274	26.438158	26.27676	27.946629	26.606129	27.2922
C02	Fabp4	16.237375	15.735806	16.033276	14.813653	15.364354	14.727357	15.517365	15.829611	16.981443	16.909185	16.140066	15.340072	15.106457	17.378235	15.843491	15.544925
C03	Fasn	16.714096	15.90506	16.458527	15.39119	15.733299	15.410765	15.799845	16.764162	18.353054	18.197931	16.70883	15.832876	15.44989	17.49945	15.822698	15.680966
C04	Fgf1	26.766582	25.798004	26.816114	26.184095	25.497688	25.726192	25.83245	27.327282	26.636385	27.847187	25.419502	26.283144	24.68085	26.767673	25.855425	25.62949
C05	Fgf10	28.24791	27.40357	27.962088	26.341978	27.934328	27.371592	27.411121	28.783052	29.593662	30.368185	27.185062	27.782387	27.504066	29.896835	28.273174	28.633722
C06	Fgf2	28.726986	28.082674	29.11495	28.44973	28.366114	28.710896	28.469212	28.865732	29.32025	30.364029	27.366446	29.091583	27.52051	28.504673	28.083017	28.400984
C07	Foxc2	30.616825	29.894741	30.58927	27.949661	30.60196	28.721518	29.861216	31.919033	32.78912	33.9837	31.746902	29.778446	30.378448	31.983309	30.421467	30.285944
C08	Foxo1	23.484392	23.121601	23.04348	22.249752	23.445013	22.757282	23.052868	23.235613	24.979702	24.74222	23.820257	22.414476	22.72084	24.20683	22.947527	23.045158
C09	Gata2	25.73076	26.284231	26.362137	25.875702	26.588171	26.132925	26.180464	27.42372	27.87351	28.524971	27.269793	26.673655	25.980593	27.794127	26.62351	26.600685
C10	Gata3	26.909464	26.610636	27.246489	24.824575	26.633564	25.590796	26.317183	27.369339	27.642382	30.21046	25.460575	26.220984	26.53954	28.638712	27.13665	26.904013
C11	Hes1	25.896915	26.661741	27.080584	26.626078	26.664436	26.892725	26.45451	27.219826	27.13829	28.180325	26.32921	27.530308	26.758305	27.238798	26.828672	27.375113
C12	Insr	25.076769	24.798923	24.86727	23.889587	25.240854	24.523754	25.030462	25.794445	26.686272	26.868982	24.88363	24.749336	24.673656	26.332981	25.248503	24.919542
D01	Irs1	24.586147	24.958132	25.955126	24.805786	23.986235	24.652975	24.435734	26.254055	26.617329	27.484566	25.379631	26.3836	24.885616	26.984428	25.817354	25.40799
D02	Irs2	22.975409	24.043762	24.925512	24.140034	23.172955	22.996283	23.531572	24.694244	25.499262	25.729816	24.821451	24.915405	24.78909	26.083427	26.656027	24.711473
D03	Jun	23.63841	25.213272	25.459875	24.762285	24.726154	24.598581	25.130024	26.622755	26.400066	27.622946	25.600939	25.713451	25.242609	26.495993	26.610023	25.666271
D04	Klf15	25.501368	25.747381	25.19843	23.877127	25.88861	24.541239	25.438803	25.281786	27.326372	27.169188	24.965601	24.486813	25.525976	27.656513	25.641586	24.888172
D05	Klf2	24.64737	25.337465	25.563297	25.351553	25.50117	25.675362	25.558691	26.275642	26.793438	27.412636	25.872734	26.217905	24.977596	27.435095	26.319561	26.030434
D06	Klf3	24.213615	24.161814	24.229355	23.426317	24.64895	24.299395	24.3844	24.633331	25.8868	26.410694	24.757912	24.308758	24.284058	25.782722	24.796335	24.344137
D07	Klf4	23.490656	23.815176	24.047691	23.85394	23.687178	23.572891	23.821556	24.658607	24.277079	25.353033	25.173952	24.38112	24.015245	25.45843	25.761187	24.376743
D08	Lep	27.4774	27.471376	27.97759	28.080227	28.503956	29.104227	28.151834	30.371	29.956348	31.825727	28.173231	30.412447	26.808683	27.777643	27.29905	30.224293
D09	Lipe	20.923908	20.387318	20.68441	19.358004	20.043903	19.658815	20.160585	21.053507	22.525082	22.633127	20.689707	20.227734	19.615002	22.075089	20.44447	20.044182
D10	Lmna	24.351912	23.985987	24.118013	23.413265	23.803923	23.171932	23.780075	24.4828	24.879635	25.717373	24.04769	23.742716	23.909565	25.085938	24.55756	23.906157
D11	Lpl	19.338224	19.212015	19.365316	17.999086	19.180548	18.005136	18.800524	18.982769	20.35392	20.081806	19.301104	18.272923	18.572548	20.831745	19.55892	18.707573
D12	Lrp5	24.937092	25.101448	24.9457	24.268274	25.44765	24.72968	25.236113	26.067083	26.480984	27.092716	25.3082	24.76988	24.614147	26.821226	25.347479	24.978512
E01	Mapk14	25.636667	25.402128	25.88817	24.531544	25.610731	24.757595	25.374788	25.813215	26.900976	27.661423	25.534418	25.294724	24.729303	26.751621	25.693254	25.260933
E02	Ncoa2	25.694094	25.362957	25.97551	25.022009	25.656588	25.619211	25.595722	26.119642	27.149973	27.75243	25.697495	25.93165	25.303732	27.096588	26.133154	25.76742
E03	Ncor2	24.990988	25.573736	25.956045	25.539091	26.016172	25.829494	26.04978	26.061863	26.866955	27.498453	25.24566	25.725876	24.926916	26.65435	25.37032	25.79935
E04	Nr0b2	27.141762	26.527979	26.772528	25.130814	26.949224	26.34267	26.175951	28.978039	29.604546	29.180994	27.970387	26.2	26.332842	28.064093	26.74781	27.177168
E05	Nr1h3	23.67422	23.61676	23.664223	22.823133	23.70481	22.762909	23.515413	23.542418	24.497862	24.542133	23.990849	23.112854	22.935524	24.691502	23.821665	23.366678
E06	Nrf1	26.259916	25.5803	26.29218	24.503803	26.32975	25.37244	25.936758	26.696562	28.251804	28.51731	26.877563	25.962791	25.940876	27.367207	26.47158	26.488398
E07	Ppara	23.209902	22.39688	22.769018	21.238651	22.30096	21.900883	22.30474	23.290588	24.7416	24.521902	23.120281	22.085926	23.16263	25.963942	24.395939	22.006859
E08	Ppard	26.932251	26.296747	26.71631	25.001049	26.954798	25.781149	26.405931	27.47029	29.085823	29.312084	27.148474	26.072659	26.19367	29.309105	27.255953	26.488749
E09	Pparg	22.12549	21.82168	22.111536	20.857931	21.915781	20.876934	21.890251	22.14867	23.277966	23.394987	22.49766	21.341608	21.320229	22.89493	21.814415	21.480766
E10	Ppargc1a	21.663797	21.7731	22.950287	21.778706	21.769142	20.777428	21.781643	22.457441	24.519367	24.129053	23.679193	22.378237	22.289762	24.29248	24.067478	22.233662
E11	Ppargc1b	22.845415	23.051632	23.173803	22.409544	22.781675	22.42489	22.925928	23.324762	23.313667	23.788937	23.48857	22.362297	22.49544	24.659369	23.087105	22.520082
E12	Prdm16	25.717615	25.260912	25.14148	24.16278	25.425266	24.53162	25.284203	25.952618	26.687822	27.095455	25.885582	24.644135	24.694227	26.544086	25.55035	25.147371
F01	Rb1	25.154932	25.019346	25.293102	24.206709	25.211504	24.729305	25.058796	25.804148	26.84487	27.17542	25.426714	25.247547	24.865877	25.745731	25.153446	25.157799
F02	Retn	26.923342	26.144873	26.52175	24.9463	26.94641	26.098763	25.790585	29.51666	29.743935	29.262285	26.866463	26.624907	26.359127	28.37525	26.817335	27.461836
F03	Runx1t1	27.926514	27.143187	28.096989	25.962013	27.95655	27.002247	27.156256	29.5097	29.779104	30.398779	28.16438	27.497795	27.59606	29.460543	27.927448	27.970379
F04	Rxra	25.392958	25.303432	25.30902	24.20425	25.500374	24.727451	25.221733	26.485386	26.915327	27.302238	25.27803	25.089424	25.015284	26.885832	25.482609	25.181124
F05	Sfrp1	29.134155	29.603968	30.101646	29.403807	30.061739	29.908667	29.90397	30.306479	30.942928	31.401571	28.721416	29.654478	29.656256	30.831944	29.592628	29.289143
F06	Sfrp5	29.314672	29.466513	29.409882	30.197521	29.748339	29.06475	29.552773	31.644638	30.1181	31.330288	28.144184	30.446842	28.917702	31.124252	29.877731	29.767532
F07	Shh	33.383392	31.490273	32.508026	29.99502	32.404694	31.088953	31.660831	32.728775	34.4693	34.338333	33.669918	31.451937	31.910975	33.50356	31.920332	32.50757
F08	Sirt1	26.39601	26.35286	26.648973	25.317083	26.439854	25.386255	26.060303	26.110477	27.257227	27.793236	26.510178	25.92949	25.914824	27.733776	26.742323	26.206697
F09	Sirt2	24.204878	23.833633	24.169699	23.075356	23.93314	23.217115	23.896105	24.35676	25.351728	25.799522	24.283855	23.724745	23.53825	25.381212	23.92291	23.769073
F10	Sirt3	23.687117	23.067366	23.322859	22.142986	23.090998	22.235952	22.744291	23.338467	25.034912	24.80514	23.927477	22.70656	22.87419	25.106525	23.424923	22.836529
F11	Slc2a4	22.723501	21.888601	22.731808	21.21025	21.753265	21.331392	21.825142	22.857117	24.095194	24.571127	22.343937	21.649702	20.976757	23.322632	21.654163	21.575506
F12	Src	29.51124	29.715126	29.218859	29.237291	29.49607	29.3078	29.511145	30.229097	31.165266	30.791723	29.684248	29.635515	29.556509	29.821205	29.657429	30.044786
G01	Srebf1	22.88383	22.800297	23.461891	22.491634	22.720211	22.08596	22.584888	22.990103	24.489199	24.607471	22.908878	22.729479	21.829794	23.69576	22.028978	22.457348
G02	Taz	20.28111	20.536453	20.707296	20.233828	20.256147	20.519281	20.31293	20.361876	22.033833	22.617495	20.885565	21.12166	20.29628	22.128668	21.26224	20.781385
G03	Tcf7l2	25.379295	25.315008	25.654116	24.824108	25.160994	24.883785	25.125578	25.536766	26.000416	26.418182	24.970114	25.312588	24.712927	25.907467	25.244223	25.28166
G04	Tsc22d3	24.607628	24.377398	24.255201	23.527857	24.630306	23.901009	24.09581	23.647999	25.292458	25.453123	23.69653	23.611368	23.937206	25.354618	23.93955	23.892653
G05	Twist1	27.198141	26.234447	27.091345	26.07088	26.944706	26.42538	26.648132	27.181051	28.322214	28.575125	26.756344	26.66021	26.230148	28.36166	27.27605	26.839027
G06	Ucp1	17.001694	16.975607	17.06469	16.07204	17.223356	15.8604	16.923912	16.920444	17.245878	17.240118	17.640005	15.865511	15.702707	17.707533	16.528778	16.310402
G07	Vdr	28.092924	28.734999	29.086298	27.615162	28.43369	27.836096	28.35749	29.896166	29.841686	30.810156	29.110846	28.770184	28.153807	30.061089	29.216331	28.816643
G08	Wnt1	32.285694	31.329128	32.300377	29.970514	31.435968	30.60625	31.350098	33.824745	34.186527	34.169086	33.457676	31.47471	31.610836	32.06692	31.721094	31.95298
G09	Wnt10b	31.078472	30.188105	31.246048	28.841358	30.58879	29.480116	30.448458	31.621279	32.76791	32.835262	31.668766	30.17119	30.456364	31.647337	31.439808	30.49219
G10	Wnt3a	32.22221	31.544928	31.83831	29.853882	31.962076	30.683136	31.445074	33.909615	37.37034	34.60331	33.013397	31.466787	31.59446	33.034508	31.838081	32.097084
G11	Wnt5a	27.31297	26.519175	27.347635	26.075146	27.154396	26.658035	26.846117	28.086838	28.357727	29.356949	26.75201	26.941555	26.397167	28.081648	27.597187	26.956228
G12	Wnt5b	31.451618	31.441965	32.69966	30.23902	31.088139	30.397951	30.97379	31.963982	31.115147	32.702984	29.68684	31.646261	31.14518	32.863	31.679491	31.355753
H01	Actb	20.55983	20.647749	20.864107	20.009537	20.51559	20.632631	20.886395	20.666895	22.211275	22.911015	20.78739	20.713123	19.975132	21.673325	20.60001	20.698927
H02	B2m	19.432014	19.3852	19.64686	19.014181	19.365784	19.548431	19.32972	19.162474	20.587934	21.306505	19.420988	19.859636	19.242296	21.135645	20.202892	19.657612
H03	Gapdh	19.200626	18.075167	18.403467	17.301733	17.687292	17.372047	17.858263	18.714287	20.21554	20.59531	18.137661	17.728935	17.311792	19.733274	18.289335	17.489048
H04	Gusb	25.97218	25.654537	26.134554	25.02197	25.859262	25.034893	25.684002	25.428343	26.784044	26.95013	25.833359	25.310364	25.060835	27.505037	26.349178	25.498123
H05	Hsp90ab1	19.992012	19.700909	19.747465	18.780836	19.653292	18.997341	19.600628	19.805294	21.446804	21.494	20.348244	19.677391	19.73334	21.442482	20.321724	19.862051

Supplemental Table 2: WAT adipogenesis PCR array Ct values

Well	Symbol	Ground ctl #33	Ground ctl #35	Ground ctl #37	Ground ctl #38	Ground ctl #39	Ground ctl #40	Flight #23	Flight #24	Flight #26	Flight #27	Flight #28	Flight #29
A01	Acacb	25.914785	27.022198	26.325565	27.718788	28.339878	27.269306	27.705471	26.489044	26.727058	27.227451	25.8095	25.38665
A02	Adig	22.865719	23.752766	22.764	24.129812	24.770779	23.707129	25.425806	23.931131	25.37125	24.647284	24.458633	23.914782
A03	Adipoq	20.953014	21.117342	20.511137	21.90436	22.524416	20.959171	23.51612	22.417099	23.971262	22.71624	22.869368	21.990948
A04	Adrb2	28.695105	29.982672	29.196722	29.785234	29.70007	29.126036	30.63754	30.035318	30.807968	29.847303	30.686375	29.722584
A05	Agt	25.818481	25.290869	24.488356	26.390545	26.729185	23.914766	27.351484	27.053524	27.675344	25.367798	26.792315	26.072393
A06	Angpt2	25.15218	27.248035	26.11745	27.131006	27.31065	26.83768	29.475332	28.558802	29.668337	28.222378	27.933973	28.20291
A07	Axin1	27.847132	29.419722	28.011848	29.12053	29.840485	28.207525	29.386095	29.415	30.737148	29.660364	29.701576	28.856842
A08	Bmp2	29.277311	31.064312	29.850992	30.086828	32.125893	30.857868	30.214895	29.96916	30.508902	30.632534	31.528507	29.973333
A09	Bmp4	28.601954	30.876627	27.770893	29.566925	29.894388	28.697695	30.61043	29.593136	33.85944	31.24041	30.726795	29.473595
A10	Bmp7	29.325687	31.211855	30.529337	31.400742	31.45819	30.556301	30.424139	29.437115	29.710535	31.18027	31.659319	29.95588
A11	Ccnd1	26.871746	27.963861	27.24838	27.968243	28.586256	27.205013	29.168835	27.997108	28.441168	28.748663	27.578661	27.740328
A12	Cdk4	26.723438	27.879393	26.841333	27.817125	28.651077	26.927692	29.222235	27.759203	29.090147	29.17401	28.65094	28.111946
B01	Cdkn1a	25.640953	26.744864	26.666515	27.460106	28.661001	26.565296	28.442528	26.646887	27.10708	26.62164	25.928802	26.230053
B02	Cdkn1b	27.783216	28.674973	27.609518	28.448536	29.314884	27.334969	29.461557	28.654976	30.162544	29.265446	29.510216	28.53509
B03	Cebpa	26.082863	26.206896	25.04178	25.960938	26.520687	26.29773	28.441698	27.829733	27.705353	25.759998	28.370018	26.68497
B04	Cebpb	28.573751	29.941423	28.805208	29.866219	30.634033	29.91956	30.288044	29.609974	30.623507	30.233795	30.10474	28.793947
B05	Cebpd	28.74746	29.223558	27.98864	28.986513	29.652248	28.596436	30.447737	30.15803	30.082636	29.297441	30.90376	29.069403
B06	Cfd	18.328999	19.454815	18.198519	19.252413	19.343124	19.785404	20.261852	19.1069	19.671139	19.176615	19.06589	18.853462
B07	Creb1	27.432753	28.673294	28.383917	28.848965	29.37865	27.883425	29.453629	28.754845	29.699902	29.219156	28.786808	28.434933
B08	Ddit3	26.905834	27.912794	26.562641	27.664179	28.927753	27.019384	29.288849	28.727057	28.787006	28.611418	28.26585	27.943117
B09	Dio2	29.10135	33.986347	33.17169	31.2177	31.73039	32.271347	30.219357	29.95089	31.53336	32.57601	32.436443	28.853874
B10	Dkk1	29.577513	33.142937	32.341114	30.826918	33.068085	Undetermined	30.346466	30.264326	36.14966	31.722654	Undetermined	29.6153
B11	Dlk1	29.243336	32.661198	29.557348	31.322529	33.254326	30.371792	30.205492	29.336031	33.784245	32.127304	32.40711	28.538622
B12	E2f1	26.994831	28.466885	27.653322	28.668055	29.942095	28.117273	28.521128	28.526974	29.988295	29.492386	29.423483	27.920181
C01	Egr2	28.555302	30.638184	30.422113	30.815062	30.778069	29.763113	29.324642	29.841316	32.358463	31.194496	32.882607	29.100517
C02	Fabp4	17.412338	17.768877	17.08606	18.529236	19.21778	17.888287	20.27516	18.786137	19.839874	19.19508	18.80968	18.545795
C03	Fasn	19.4307	20.670715	19.182041	20.587488	22.019407	22.043142	21.798334	20.233469	20.816828	21.646456	20.290257	19.9001
C04	Fgf1	27.31699	28.403341	25.659832	29.40403	30.09972	28.084227	30.165272	28.918583	29.688456	29.692781	28.344294	28.59047
C05	Fgf10	25.430412	26.47102	25.961237	26.872805	27.235502	26.681355	28.426247	27.183243	28.390112	27.611557	27.691717	26.996338
C06	Fgf2	27.44976	28.340082	27.395243	28.599726	29.172216	27.113474	29.239185	28.867472	31.430674	29.25544	30.808338	28.484316
C07	Foxc2	29.676676	32.209343	30.976387	31.593697	32.622173	31.7514	31.830173	30.451817	33.681454	32.609013	32.80651	30.258778
C08	Foxo1	26.007664	26.52206	25.816368	26.607471	27.642956	25.915152	28.389368	27.136753	28.280308	26.601765	27.432062	26.902754
C09	Gata2	27.981993	30.142942	28.242924	29.329643	29.565365	29.51128	29.75926	28.948677	28.717836	29.420084	30.544127	28.77216
C10	Gata3	28.348198	31.023579	28.616467	29.629353	31.788723	28.85854	28.927994	26.841501	27.33781	30.798674	30.96908	29.257298
C11	Hes1	28.785835	30.104336	29.826694	29.726606	31.397024	30.089085	30.939306	29.273533	28.939238	30.41975	30.659426	29.854118
C12	Insr	26.683517	27.528952	26.40383	27.415693	28.735796	26.760637	28.733366	27.998016	29.114634	27.942835	28.53418	27.979729
D01	Irs1	25.741873	27.35013	26.454704	27.480263	28.473768	26.980467	28.464024	27.61375	29.868996	28.481216	28.707386	28.11462
D02	Irs2	27.1182	26.96049	25.655003	27.29774	27.818695	26.851833	28.661718	28.695532	29.60893	27.911795	29.49749	28.662327
D03	Jun	26.3166	27.567883	25.240816	26.751814	27.924412	26.58478	29.884756	28.346062	29.608772	27.538181	29.429832	28.293852
D04	Klf15	26.158155	26.361616	25.69091	26.876026	27.662428	25.732954	28.682991	27.622574	29.790783	27.24696	28.236158	27.519821
D05	Klf2	27.314344	28.41286	26.009876	27.775448	28.427969	26.833803	29.999249	29.112078	30.092278	28.577848	29.291357	28.443363
D06	Klf3	26.353954	27.31205	25.296438	26.611973	27.684717	26.312685	29.275843	27.892214	27.939262	27.429441	27.899841	27.384993
D07	Klf4	27.263292	27.820604	26.829397	28.506138	28.492338	27.218517	29.730865	28.846848	29.281454	28.771442	28.748299	28.37155
D08	Lep	25.605043	24.93048	24.176516	25.651934	27.012756	23.961643	29.38285	28.532001	27.098932	25.538498	26.605204	25.94465
D09	Lipe	22.644228	22.56653	21.697636	23.505953	24.085554	22.36715	24.876162	23.957973	25.38758	23.803747	24.235888	23.369032
D10	Lmna	26.322882	26.82214	25.735258	27.136028	27.845339	26.242575	28.74259	27.599445	27.969353	27.576225	27.462122	27.225393
D11	Lpl	20.130596	20.284475	19.86765	20.852781	22.07871	20.506071	23.386677	21.817781	23.10078	21.818846	22.145811	21.535284
D12	Lrp5	26.70206	27.429573	26.104113	27.55033	28.59022	26.833237	29.859894	28.679249	29.09427	28.20768	28.066563	27.52187
E01	Mapk14	26.702887	27.736147	26.903772	28.001583	29.047361	26.870316	29.312977	27.974829	28.643194	28.300425	28.036768	27.332418
E02	Ncoa2	27.508389	28.308973	26.98996	28.015583	29.175781	27.569574	30.086859	28.65806	29.425655	28.574263	29.32625	28.269327
E03	Ncor2	27.483236	28.349554	26.111898	27.157244	28.882992	27.741055	30.112257	28.81117	29.196543	27.930456	28.583715	27.812218
E04	Nr0b2	28.108244	29.31386	29.399616	29.253832	29.393873	29.371414	29.354538	29.795856	30.516224	29.972933	27.754368	28.782324
E05	Nr1h3	26.112509	26.37014	25.50303	26.851667	27.357872	26.42014	28.15771	27.208406	28.141092	26.852205	26.964884	26.735245
E06	Nrf1	27.521915	29.08639	28.428638	28.865267	29.042244	28.495655	28.73785	28.67107	29.508522	29.422945	29.23423	28.425276
E07	Ppara	28.959293	30.231022	29.336893	30.51577	30.64603	29.522564	29.705309	29.666796	31.509392	31.076862	32.527565	29.084742
E08	Ppard	29.048037	29.964903	28.91207	30.901312	31.196768	29.55277	30.772121	30.304018	30.804459	30.73394	30.348364	29.585823
E09	Pparg	24.39311	24.943834	23.86002	24.953247	26.002548	24.494953	27.100657	25.692623	26.350895	25.521154	25.393293	25.30975
E10	Ppargc1a	28.360903	30.260452	28.870497	29.533327	31.291039	28.77769	29.181293	29.13628	31.534235	30.67974	30.271223	28.763771
E11	Ppargc1b	28.590515	29.913433	28.250093	29.301308	30.341246	29.567165	30.002134	28.83581	30.519972	30.514967	28.577555	28.835556
E12	Prdm16	27.925539	29.713863	29.457829	29.795769	30.179535	29.637854	29.340097	29.319849	30.368532	30.334316	30.147312	29.13043
F01	Rb1	26.554354	27.204464	26.615667	27.744469	28.38823	26.652977	29.37118	28.140743	29.177788	28.408035	28.290968	27.657068
F02	Retn	25.998407	27.82266	26.805805	27.668472	28.116898	27.359867	28.302383	27.470724	29.1532	28.117985	27.73486	27.526148
F03	Runx1t1	28.669394	30.629642	29.357065	30.492088	31.398882	29.624231	29.58318	29.492987	30.8824	30.80981	29.529627	28.674482
F04	Rxra	26.575663	26.791552	25.717958	26.931911	27.718493	26.626274	28.63829	27.75361	27.518606	26.6756	27.769104	26.97197
F05	Sfrp1	28.828688	30.65822	30.164919	28.563448	29.58248	26.995298	30.484283	29.578476	30.967983	33.400646	30.823399	28.362484
F06	Sfrp5	28.912634	29.339441	27.133474	29.753633	33.22714	27.538586	30.306738	29.705538	31.812374	29.114498	31.665768	29.741518
F07	Shh	29.956697	33.899525	32.04185	31.487608	34.273193	33.08235	31.317127	30.331812	31.491903	32.311363	34.089043	30.011404
F08	Sirt1	26.992664	28.449183	27.941227	28.881256	29.940027	28.16701	29.880457	28.935682	29.955982	29.316498	29.423738	28.258696
F09	Sirt2	26.74807	27.423489	26.56116	27.803118	28.09395	26.65617	29.001728	27.81175	29.1258	28.456575	28.116585	27.715387
F10	Sirt3	27.407738	28.621668	27.911	29.052326	29.568436	27.558384	28.578548	28.3445	29.534058	29.217775	29.392141	27.914131
F11	Slc2a4	24.997868	25.523283	24.985292	26.078405	26.804762	25.120169	26.986744	25.859127	25.981258	26.259138	25.236874	25.038685
F12	Src	29.336334	30.118935	29.18894	29.930573	30.515419	29.733871	30.07285	29.85115	30.22175	30.25353	30.724083	29.638288
G01	Srebf1	24.655764	25.511072	24.652796	25.644444	26.37144	25.647266	26.564207	25.656374	25.992495	25.690104	24.914665	24.53863
G02	Taz	27.08559	27.708918	27.495144	28.217722	28.325922	27.247927	29.065224	28.356955	28.861317	28.963924	28.542858	27.806833
G03	Tcf7l2	26.555231	27.036736	26.07319	27.178251	27.83853	26.497187	28.156338	27.301584	28.094784	27.857897	27.55288	27.170969
G04	Tsc22d3	25.28852	25.400398	25.571157	26.337458	26.92398	24.320957	28.202415	26.9439	28.415516	26.704605	26.5314	26.115814
G05	Twist1	28.319475	29.347183	28.155142	29.320189	30.376314	29.038223	30.452538	28.959875	30.989866	29.894945	29.952393	29.148438
G06	Ucp1	29.525948	32.196903	31.391367	33.400814	31.716341	34.10568	29.951351	30.35223	25.875912	31.096771	30.389042	24.245464
G07	Vdr	28.958776	34.31661	31.3171	32.24235	33.519524	31.899235	31.913824	29.365608	33.509354	38.00066	32.43676	29.373932
G08	Wnt1	30.091457	33.83496	32.569714	33.62358	33.001797	33.215076	31.842796	30.863894	35.659225	32.219727	35.10357	30.892103
G09	Wnt10b	29.600018	33.747753	31.996649	33.525578	33.841103	32.452587	29.791538	30.434778	33.648243	32.961884	32.96876	31.944231
G10	Wnt3a	30.168314	33.35072	33.6715	32.341873	33.224052	33.546528	31.168043	30.738518	33.295116	32.62934	33.697193	31.257696
G11	Wnt5a	27.998404	28.770689	27.529316	28.603012	29.856989	28.272284	29.837732	28.167288	28.677187	29.442612	29.194641	28.927214
G12	Wnt5b	30.59459	32.819405	31.383556	32.887245	33.294945	30.839073	31.3735	31.965452	34.635723	32.929768	32.331432	30.55871
H01	Actb	21.44333	21.740194	21.242245	22.516176	23.30461	21.143974	24.552616	23.251175	24.158901	23.204994	22.804293	22.87089
H02	B2m	19.58497	20.120472	19.397017	20.80686	21.49417	19.56818	22.531565	20.800358	21.736881	21.73711	21.178299	21.154066
H03	Gapdh	20.648863	21.914192	20.936079	22.21445	23.424839	22.108799	23.031256	21.896496	22.48656	23.22713	21.974867	21.502209
H04	Gusb	27.045542	27.422667	26.67872	27.754414	28.614157	27.138361	29.434656	28.474377	29.761122	28.638416	28.541494	27.976278
H05	Hsp90ab1	22.486605	22.917744	21.87411	23.1108	24.059973	22.494522	25.277958	23.661436	24.142675	23.636732	23.59492	23.625772
